# Intrathoracic Gastric Volvulus Mimicking a Cardiopulmonary Emergency: An Unusual but Fatal Diagnostic Pitfall in Critical Care

**DOI:** 10.7759/cureus.105496

**Published:** 2026-03-19

**Authors:** Younes Hamdi, Mohamed A Fehdi, Dafir Asmae, Badria Aggoug, Mohammed Mouhaoui

**Affiliations:** 1 Emergency Department-Trauma Center, Ibn Rochd University Hospital, Casablanca, MAR

**Keywords:** acute respiratory failure, hiatal hernia, intrathoracic gastric volvulus, obstructive shock, ‎tension gastrothorax

## Abstract

Hiatal hernias are often incidental findings, yet acute intrathoracic gastric volvulus in large hernias can rapidly evolve into catastrophic cardiopulmonary collapse. We report the case of a 65-year-old man presenting with progressive dyspnea and retrosternal pain who deteriorated into hypoxemic respiratory failure and shock. Despite initial stabilization with high-flow oxygen, fluid resuscitation, and vasopressor support, he remained hemodynamically unstable. Chest computed tomography revealed a massive sliding hiatal hernia complicated by organoaxial gastric volvulus occupying the left hemithorax with mediastinal shift and lung compression. Within hours, the patient developed sudden neurological decline and refractory shock, progressing to pulseless electrical activity despite resuscitative efforts. This case underscores intrathoracic gastric volvulus as a rare but fatal mimic of primary cardiopulmonary emergencies in the critical care setting, emphasizing the need for early imaging and urgent surgical assessment when clinical severity exceeds pulmonary findings.

## Introduction

Acute respiratory distress with hemodynamic instability is most commonly attributed to primary pulmonary or cardiac causes. However, uncommon conditions involving intrathoracic displacement of abdominal organs may lead to respiratory compromise and represent a critical diagnostic challenge.

Hiatal hernias are common and often discovered incidentally on imaging performed for unrelated indications. However, in large hiatal hernias, an acute intrathoracic migration of the stomach may result in gastric volvulus and severe cardiopulmonary compromise [1]. Gastric volvulus is an abnormal rotation of the stomach around its axis, which can lead to obstruction, impaired perfusion, and rapid clinical deterioration if not promptly recognized [2,3]. While gastrointestinal symptoms are typical, cardiopulmonary manifestations may occasionally predominate, potentially mimicking primary cardiopulmonary emergencies [4,5].

We report a case of massive hiatal hernia complicated by organoaxial gastric volvulus presenting primarily as acute respiratory distress and refractory shock, highlighting a rare but fatal diagnostic pitfall in critical care.

## Case presentation

A 65-year-old male with a history of hypertension treated with amlodipine, obesity (BMI 30 kg/m²), and former chronic smoking presented with five days of progressive dyspnea and retrosternal pain, acutely worsening on the day of admission. There was no prior history of gastroesophageal reflux, dysphagia, or known hiatal hernia.

On admission, the patient was confused with a Glasgow Coma Scale score of 14/15. Vital signs showed hypotension (79/45 mmHg), tachycardia (110 beats per minute), and tachypnea (28 breaths per minute), with oxygen saturation of 85% on room air. Chest auscultation revealed decreased breath sounds over the left hemithorax without wheezing or crackles. No bowel sounds were clearly identified on thoracic auscultation. Abdominal examination showed a soft, non-distended abdomen without peritoneal signs, guarding, or rigidity. The patient was also afebrile, and capillary blood glucose was measured at 1.10 g/L. Initial management included high-flow oxygen, intravenous fluids, and vasopressor support.

**Figure 1:**
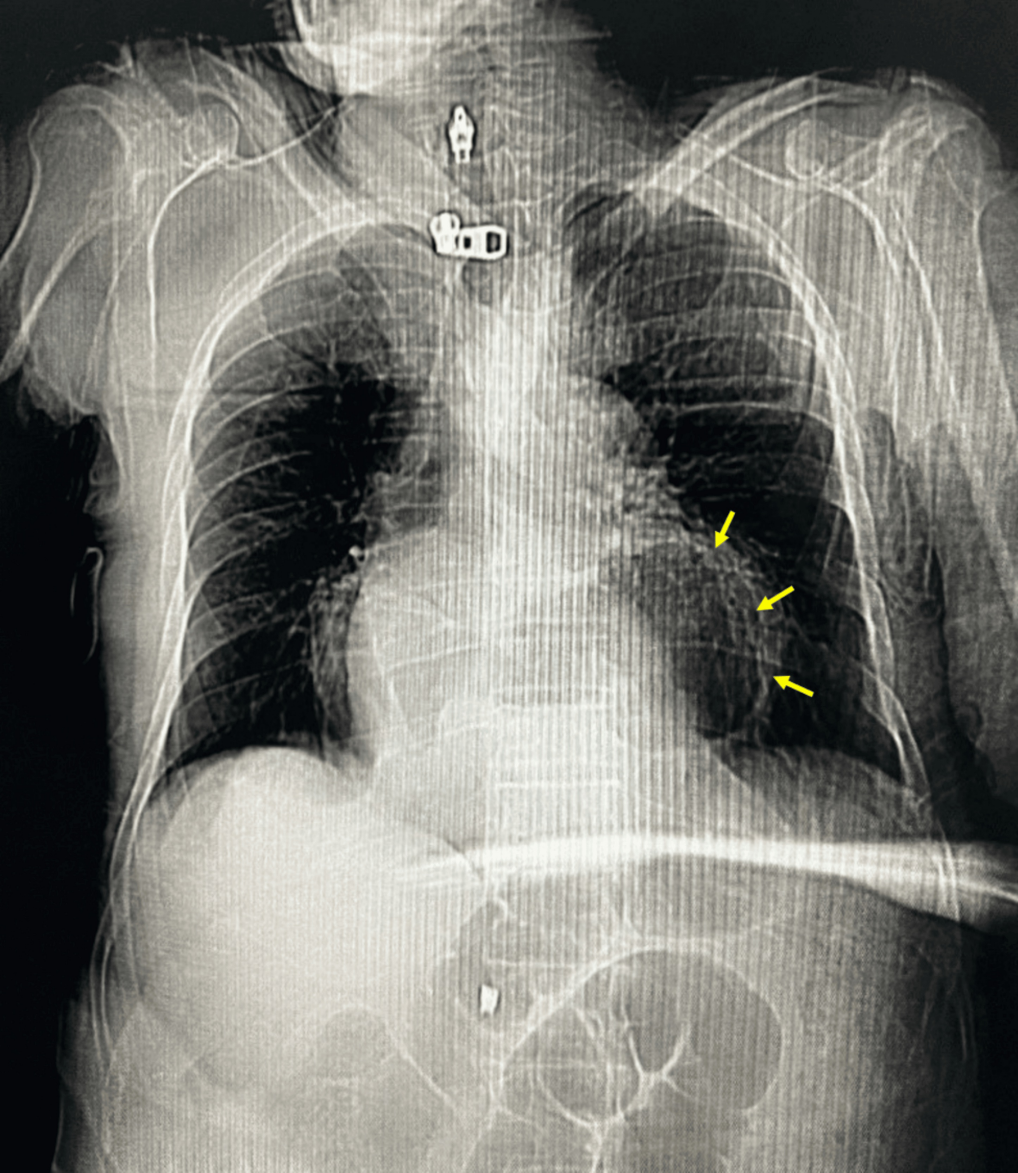
Chest scout radiograph‎ Frontal scout view demonstrating a large retrocardiac air-containing structure (arrows) occupying the left hemithorax, with rightward mediastinal shift.

**Figure 2:**
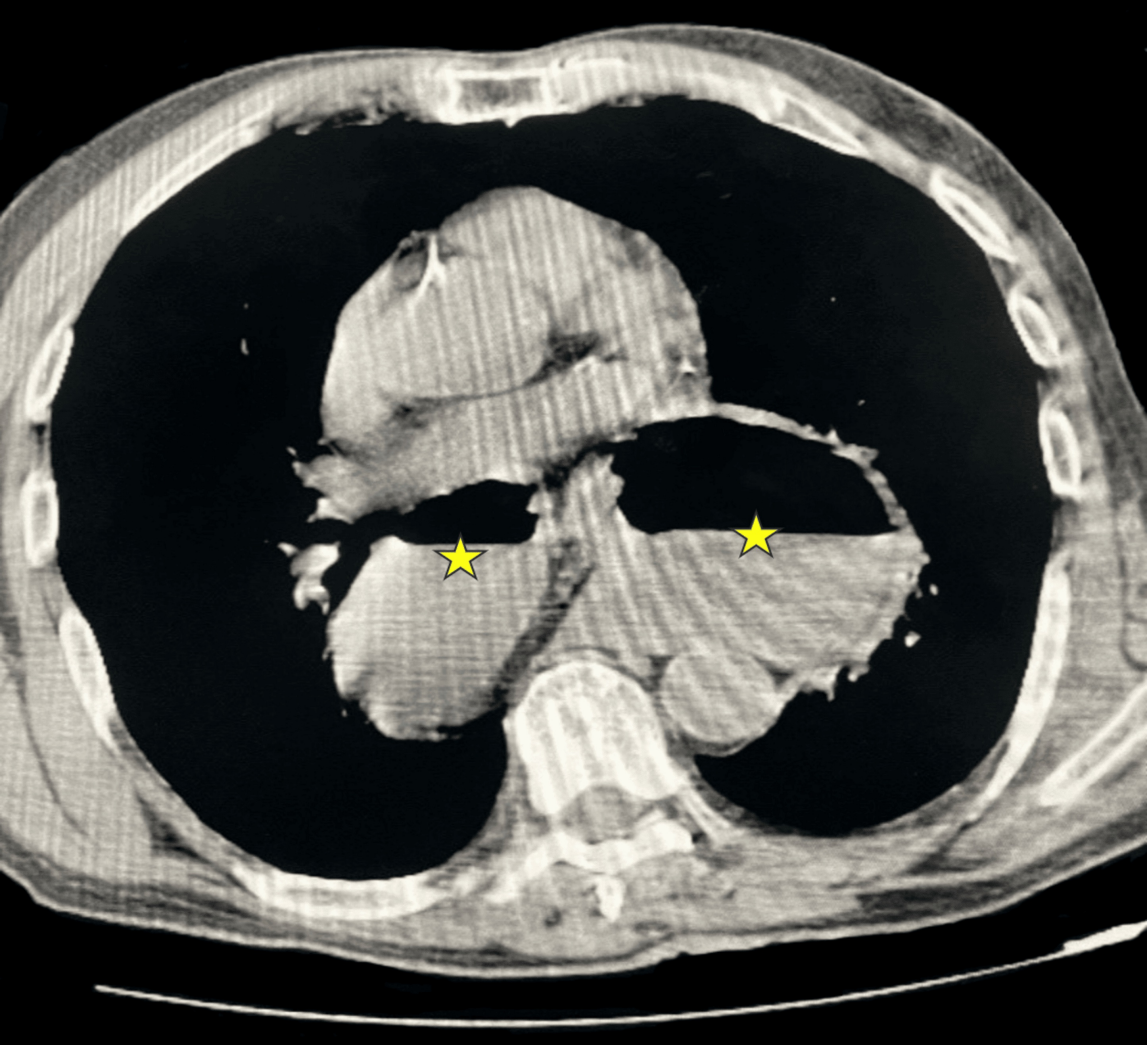
Axial thoracic computed tomography Shows a markedly distended intrathoracic stomach within the posterior mediastinum, with air-fluid levels (asterisks).

**Figure 3:**
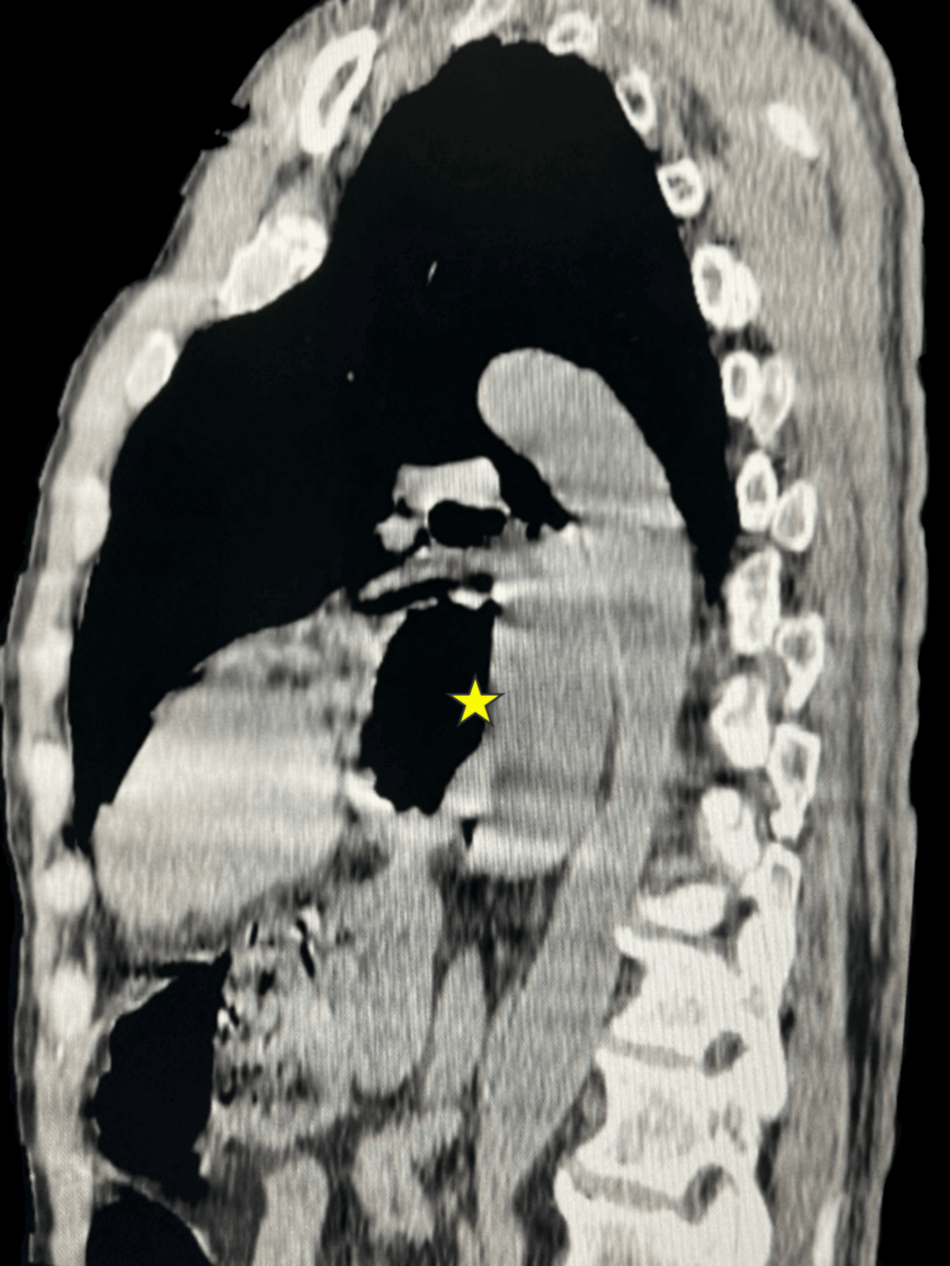
Sagittal reconstruction ‎of thoracic computed tomography ‎ Herniation of the stomach through the esophageal hiatus with abnormal gastric orientation (asterisk).

The initial differential diagnosis included pulmonary embolism, cardiogenic shock, septic shock of pulmonary origin, and acute exacerbation of chronic obstructive pulmonary disease (COPD). Point-of-care ultrasound (POCUS) was not available at the time of the patient’s initial evaluation. Electrocardiography showed sinus tachycardia without ischemic changes, and no clinical signs of infection were identified. Chest computed tomography ultimately excluded pulmonary embolism and revealed a massive hiatal hernia with organoaxial gastric volvulus occupying a large portion of the left hemithorax, associated with marked mediastinal shift and compression of the left lung (Figures 1-3). No definite signs of perforation or ischemia were identified on initial imaging. Urgent surgical consultation was obtained, and emergency operative management was planned.

Approximately two hours after presentation, rapid neurological deterioration with a Glasgow Coma Scale score of 10/15, worsening respiratory failure, and refractory shock required escalation of vasopressor support. Within minutes, the patient developed pulseless electrical activity. Advanced cardiac life support was initiated immediately; however, despite prolonged resuscitative efforts, spontaneous circulation was not restored, and the patient was pronounced dead approximately 2.5 hours after arrival at the emergency department. No autopsy was performed.

The precise mechanism of death remains uncertain, but acute cardiopulmonary failure in the setting of massive intrathoracic gastric volvulus was considered the most likely cause.

## Discussion

Massive hiatal hernia complicated by intrathoracic gastric volvulus is uncommon but potentially catastrophic. While many hiatal hernias remain asymptomatic, massive herniation into the thoracic cavity may be associated with severe complications, including gastric volvulus and cardiopulmonary compromise [1,6]. The clinical presentation of gastric volvulus can vary considerably. In our patient, respiratory distress and hemodynamic instability predominated over gastrointestinal symptoms, which likely contributed to the diagnostic delay, as previously reported [4,5,7]. Careful physical examination may provide important clues. Decreased breath sounds over one hemithorax, especially when disproportionate to the clinical picture, should raise suspicion of intrathoracic displacement of abdominal organs. In some cases, bowel sounds may even be heard on thoracic auscultation, suggesting intrathoracic herniation of a hollow viscus [2,5]. In our patient, breath sounds were decreased over the left hemithorax, consistent with the CT findings, while the abdominal examination remained unremarkable, reflecting the intrathoracic location of the pathology. Hemodynamic compromise in large hiatal hernias may occur due to cardiac compression and impaired venous return [7].

The underlying mechanism may resemble tension gastrothorax physiology: progressive gastric distension within the thoracic cavity can lead to pulmonary compression, mediastinal shift, and impaired venous return secondary to elevated intrathoracic pressure. The resulting reduction in preload may produce a form of obstructive shock that clinically mimics massive pulmonary embolism or tension pneumothorax [4,5]. Therefore, early hemodynamic assessment is crucial to distinguish distributive, cardiogenic, and obstructive shock. Persistent hypotension despite fluids and vasopressors should raise suspicion of impaired cardiac filling. POCUS, following the rapid ultrasound in shock and hypotension (RUSH) protocol, may demonstrate signs of reduced preload with preserved ventricular function; while these findings are non-specific and may overlap with distributive shock, their persistence despite fluid resuscitation should raise suspicion of an obstructive mechanism and prompt urgent cross-sectional imaging to identify extracardiac causes [8]. In our case, POCUS was unavailable, highlighting the importance of its systematic use in refractory shock of unclear etiology.

Computed tomography remains the diagnostic modality of choice in emergency settings. It enables prompt identification of herniated organs, confirmation of volvulus, and evaluation of complications [9].

Management requires a multidisciplinary approach combining resuscitation and urgent surgical planning. In the context of obstructive shock, volume resuscitation should remain cautious to avoid worsening intrathoracic pressure, while norepinephrine is preferred to maintain perfusion pressure. If mechanical ventilation is needed, a minimal positive end-expiratory pressure (PEEP) and a low tidal volume are preferred to limit further compromise of venous return [8]. Nasogastric tube insertion may be attempted as a temporizing measure to relieve intrathoracic compression, though it carries perforation risk in complete volvulus [2,10]. Definitive treatment is surgical, involving gastric reduction, volvulus detorsion, and hiatal repair with gastropexy or fundoplication [1,2]. Mortality in the emergency setting is substantially higher than in elective repair, with acute presentations carrying a particularly poor prognosis in elderly or hemodynamically unstable patients [1,6]. In our patient, rapid progression to pulseless electrical activity precluded any operative intervention, underscoring the critically narrow therapeutic window in such cases.

This case highlights the importance of maintaining a broad differential diagnosis in unexplained respiratory or hemodynamic deterioration, particularly when initial cardiac and pulmonary evaluations are inconclusive.

## Conclusions

Intrathoracic gastric volvulus is a rare but life-threatening cause of acute respiratory failure and shock that may closely mimic primary cardiopulmonary emergencies, particularly when clinical severity exceeds initial pulmonary findings. Early imaging and urgent surgical evaluation are critical to prevent irreversible hemodynamic collapse.
